# Modeling mycorrhizal fungi dispersal by the mycophagous swamp wallaby (*Wallabia bicolor*)

**DOI:** 10.1002/ece3.6873

**Published:** 2020-10-13

**Authors:** Melissa A. Danks, Natalie Simpson, Todd F. Elliott, C. E. Timothy Paine, Karl Vernes

**Affiliations:** ^1^ Centre for Ecosystem Management Edith Cowan University Joondalup WA Australia; ^2^ Environmental and Rural Science University of New England Armidale NSW Australia

**Keywords:** animal‐mediated dispersal, dispersal distance kernel, ectomycorrhizal fungi, fungal dispersal, mammal‐fungal interaction, mycophagy, spore dispersal, spore dispersal distance, truffle‐like fungi

## Abstract

Despite the importance of mammal‐fungal interactions, tools to estimate the mammal‐assisted dispersal distances of fungi are lacking. Many mammals actively consume fungal fruiting bodies, the spores of which remain viable after passage through their digestive tract. Many of these fungi form symbiotic relationships with trees and provide an array of other key ecosystem functions. We present a flexible, general model to predict the distance a mycophagous mammal would disperse fungal spores. We modeled the probability of spore dispersal by combining animal movement data from GPS telemetry with data on spore gut‐retention time. We test this model using an exemplar generalist mycophagist, the swamp wallaby (*Wallabia bicolor*). We show that swamp wallabies disperse fungal spores hundreds of meters—and occasionally up to 1,265 m—from the point of consumption, distances that are ecologically significant for many mycorrhizal fungi. In addition to highlighting the ecological importance of swamp wallabies as dispersers of mycorrhizal fungi in eastern Australia, our simple modeling approach provides a novel and effective way of empirically describing spore dispersal by a mycophagous animal. This approach is applicable to the study of other animal‐fungi interactions in other ecosystems.

## Graphical abstract

Animal‐mediated dispersal facilitates the spread of truffle‐like fungi spores, yet tools to empirically estimate spore dispersal distance are lacking. A simple modeling approach, combining animal movement and gut‐retention data, predicts the distance a mycophagous animal disperses spores. We test the model using the swamp wallaby (*Wallabia bicolor*) and find this mammal disperses ingested spores hundreds of meters from points of ingestion, greater distances than truffle‐like fungi are expected to spread vegetatively. Our study highlights the ecological importance of swamp wallabies as dispersers of mycorrhizal fungi and provides an empirical modeling approach that can be applied to other animal‐fungi interactions.

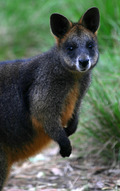

## 1 INTRODUCTION

Animal movement patterns play a role in animal‐mediated ecological processes, including propagule dispersal. Mechanisms of propagule dispersal have been widely studied, particularly among plants (Levin et al., 2003). These dispersal mechanisms are diverse and ecologically complex, which makes them challenging to measure or predict; this in turn limits the predictive capacity of dispersal models, almost all of which have been developed for seeds of plants (Aslan et al., 2019). The role of animals in fungal spore dispersal is similarly complex and ecologically important but has been far less thoroughly studied (Elliott, Bower, et al., 2019; Elliott, Jusino, et al., 2019; Fogel & Trappe, 1978; Tuno, 1998; Vašutová et al., 2019).

Fungi form a multitude of fruiting morphologies that depend on different mechanisms of dispersal. Some types discharge spores into air currents (Money, 1998; Pringle et al., 2005), others function like tumbleweeds (Bates, 2004; Kreisel, 1962). Fungi with hypogeous (below ground) and sequestrate (truffle‐like) fruiting morphologies (hereafter referred to as truffle‐like fungi) are considered dependent on animal dispersal since their spores are trapped inside their fruiting bodies (sporocarps) and/or below the soil surface (Trappe & Claridge, 2005). The spores of nonsequestrate and epigeous (fruiting above ground) species more easily end up in air currents, but while wind dispersal of these spores is effective over long‐distances, a few studies have shown the vast majority of spores land within a few meters of the fruiting body (Galante et al., 2011; Horton, 2017; Li, 2005). Truffle‐like fruiting morphologies have independently arisen in many different fungal lineages, likely due to a variety of reproductive and dispersal advantages including protection from desiccation and dispersal by animal mycophagy (Elliott & Trappe, 2018; Sheedy et al., 2016; Tedersoo et al., 2010; Thiers, 1984; Trappe & Claridge, 2005). Truffle‐like fungi are predominantly ectomycorrhizal (EM), forming associations that assist a wide variety of plants in nutrient absorption and growth (Trappe & Claridge, 2005).

Among EM fungi, dispersal limitation, and a dispersal‐competition trade‐off influence species richness and community structure (Peay et al., 2007, 2012). The availability of spores limits the effectiveness of EM fungal colonization of roots (e.g., Trowbridge & Jumpponen, 2004) and therefore reduces plant establishment (Cázares et al., 2005; Trappe, 1988). Patterns of propagule dispersal are also influenced by landscape heterogeneity (Levey et al., 2008). In contiguous forest habitats with similar species compositions, EM fungi can colonize seedlings through mycelial spread (Jonsson et al., 1999); but in areas with spatial or temporal barriers to vegetative growth, spores are the primary means of dispersal (Bruns et al., 2009). Spores are vital propagules, even for EM fungi in contiguous forest habitats (Redecker et al., 2001); spores enable genetic recombination (Kytöviita, 2000).

Long‐distance dispersal (LDD; i.e., dispersal at the scale of several meters to kilometers) is vital for transport of inoculum beyond the root‐hyphal zone of mature trees to uncolonized habitats such as isolated or regenerating forest patches (Ashkannejhad & Horton, 2005; Cázares & Trappe, 1994; Johnson, 1995; Terwilliger & Pastor, 1999). The spores of truffle‐like fungi tend to be deposited in situ (Miller et al., 1994) or transported short distances by invertebrates (Houston & Bougher, 2010; Lilleskov & Bruns, 2005; Reddell & Spain, 1991). For LDD to occur, however, vertebrate vectors such as mammals, birds, or reptiles are needed (Cooper & Vernes, 2011; Elliott, Bower, et al., 2019; Elliott, Jusino, et al., 2019; Fogel & Trappe, 1978; Luoma et al., 2003). Animal vectors of spore dispersal (particularly those traversing habitat boundaries) are crucial to assisting truffle‐like fungi in the colonization of associated plants in naturally patchy habitats, modified landscapes or “new” habitats such as glacial forefronts (Cázares & Trappe, 1994), beaver meadows (Terwilliger & Pastor, 1999), and stabilizing sand dunes (Ashkannejhad & Horton, 2005).

Many spores remain viable after passage through the mammalian gut and, if deposited in suitable locations, will go on to germinate and form mycorrhizal associations (Colgan & Claridge, 2002; Kotter & Farentinos, 1984; Piattoni et al., 2014; Reddell et al., 1997; Tay et al., 2018; Trappe & Maser, 1976). Gut passage may even enhance spore viability and rate of mycorrhizae formation (Caldwell et al., 2005; Claridge et al., 1992; Lamont et al., 1985) or seedling growth (Dundas et al., 2018; Valentine et al., 2018). Dundas et al. (2018) showed that seedlings grown in soil intensely dug by mycophagous mammals exhibit greater above‐ground biomass and significantly different rhizosphere fungal community composition, including a greater proportion of putatively truffle‐like EM fungi, compared to seedlings grown in soil less intensely dug by mycophagous mammals. Spores deposited in feces enter the root zone through water infiltration (Maser et al., 1978; Trappe & Maser, 1977) or the digging activities of animals (Claridge et al., 1992; Fleming et al., 2014) and then come into contact with the fine roots of potential host plants.

Landscapes dominated by EM host plants often support diverse mycophagous mammal communities. For example, the woodlands and forests of southern temperate Australia, which are dominated by *Eucalyptus* spp, are home to at least 30 species of mycophagous marsupials and rodents (Claridge & May, 1994; Nuske et al., 2017). These co‐occurring mammals are important players in a complex system of animal‐fungi‐plant interactions that help to maintain healthy ecosystems. Some mycophagists are specialists, relying primarily on fungi for nutrition, and these include six of the eight potoroids (family Potoroidae; Claridge & May, 1994; Nuske et al., 2017). The majority of mammals that consume fungi are generalist mycophagists; they consume varying amounts and diversities of sporocarps along with other food items (Claridge & Trappe, 2005; Nuske et al., 2017). In mycophagous mammal communities, species tend to vary their feeding habits between seasons, different habitats, and different fungal species; spore dispersal thus occurs at different scales depending on the time and place of consumption (Claridge & May, 1994; Pyare & Longland, 2001; Tory et al., 1997; Vernes, 2007, 2010). Many different mycophagous mammals may therefore contribute to the maintenance of local fungal communities and genetic mixing of fungi populations. Specialist mycophagists consistently consume and disperse a greater diversity of fungal spores (Nuske et al., 2017), but both specialist and generalist mycophagists play important roles in maintaining diverse fungal communities and therefore diverse and healthy ecosystems. These associations have been relatively well studied in Australia, but there are still many other regions of the world where there is little or no data about the role vertebrates play in the dispersal of fungi. As more information becomes available about the diets of previously understudied animals, we can begin to empirically model animal‐mediated spore dispersal. Modeling dispersal of fungi will lead to a better understanding of fungal biogeography/evolution and the dispersal advantages of fruiting morphologies that are readily detected and consumed by animals.

This study uses a straightforward and easily replicable approach, similar to those used to estimate propagule dispersal distance in studies of animal‐mediated seed dispersal (Levin et al., 2003; Nathan et al., 2012). We chose the dispersal distance modeling approach for its relative simplicity, wide use in the seed dispersal literature, and ease of application to animal‐mediated spore dispersal. Using the swamp wallaby (*Wallabia bicolor*) as an example, we directly relate empirical gut‐passage data and animal movement data to predict the distribution of distances to which spores are likely to be transported from the point of consumption. The swamp wallaby was selected because its gut‐retention time has previously been established for the spores of the truffle‐like fungi it ingests (Danks, 2011, 2012), and its stable population allows for live‐trapping to monitor movements. Unlike many of the smaller mycophagous species, *W. bicolor* has maintained substantial population numbers despite the introduction of exotic predators (Fleming et al., 2014) and is one of the most common and widespread native mycophagous mammals in eastern Australia (Claridge et al., 2001; Danks, 2011; Hollis et al., 1986; Osawa, 1990; Vernes & McGrath, 2009). We estimate the dispersal distance of spores ingested by this generalist mycophagous mammal and provide a novel approach that can be utilized by future researchers to model spore dispersal by vertebrates at landscape scales.

## 2 MATERIALS AND METHODS

### 2.1 Animal movement data

Movement data were collected on the swamp wallaby (*W. bicolor*) at Newholme Field Laboratory (30.41°S, 151.64°E, 980–1,390 m a. s. l.) and Booroolong Nature Reserve (30.33°S, 151.58°E, 1,170–1,330 m a. s. l.) near Armidale in the New England bioregion of New South Wales, Australia. The vegetation cover of the study sites is typical of the bioregion: a mosaic of eucalypt woodland, forest, and natural grasslands, patchily cleared of native vegetation with remnant forest, remnant woodland, and isolated trees scattered throughout cleared areas (McIntyre & Barrett, 1992). The mammal community at the study site and through much of Australia has been greatly simplified due to invasive predators. Many of the fungal specialists such as potoroids and native rodents have been extirpated or are in low enough numbers to be functionally extinct. The swamp wallaby is one of a small number of vertebrate mycophagists remaining that consumes a diversity of truffle‐like fungi at a frequency and diversity comparable to that of fungal specialists (Nuske et al., 2017).

A total of six adult swamp wallabies were captured and tracked between 2008 and 2016: one female and three male wallabies between February and August 2008 and an additional two male wallabies between July and October 2016. Wallabies were trapped with custom‐built soft‐walled traps, then fitted with custom‐built global positioning system (GPS) telemetry units. GPS telemetry units consisted of a GPS logger (either iTrax03; Fastrax, or I‐gotU GT‐I20; Mobile Action Technology) packaged with a two‐stage very high frequency (VHS) transmitter module with inbuilt battery (RI2CM; Holohil Systems Ltd.). The units weighed 65–75 g (<1.5% of body weight) and were attached to a small area of clipped fur between the shoulder blades using quick‐setting surgical adhesive. Telemetry units were programmed to record positional information every 15 or 30 min and designed to detach from the wallaby within several weeks as the glue bond weakened; units emitted an increased VHS signal pulse (stationary mode) when their position had not changed for 12 hr. The VHF signal was regularly checked using a Telonics TR2 portable receiver and 3‐element folding Yagi antenna. Telemetry units signaled stationary mode when they had detached from the animal; units were then collected and the GPS data retrieved. The six wallabies were tracked for a total of nine tracking periods; two wallabies were tracked more than once during the study. No ill effects were observed in wallabies after having worn a telemetry unit. Traps were closed during each tracking period to avoid interference with normal foraging behavior. To improve location accuracy, position information was recorded only when at least four satellites were visible (i.e., 3‐dimensional fixes). A locational accuracy study by Danks (2011) found that the mean error radius of 3‐dimensional location fixes recorded by units containing iTrax03 loggers was 14.6 m. Animal capture and handling protocols followed guidelines of the American Society of Mammalogists (Gannon & Sikes, 2007; Sikes & Gannon, 2011) and was approved by the Animal Ethics Committee at the University of New England (codes AEC07‐191, AEC09‐023, and AEC16‐038). Further details of movement data collection methods are found in Danks (2011) and Simpson (2016).

### 2.2 Fungal spore gut passage

Danks (2012) examined the passage time of fungal spores through the digestive system of swamp wallabies by observing the defecation rate of marker fungal spores in two adult male swamp wallabies. In that study, two wallabies were held under semi‐natural conditions at Newholme Field Laboratory in the New England bioregion of New South Wales, Australia. After an acclimation period of 3 days, the wallabies were fed a slurry containing marker spores, the distinctive spores of the ectomycorrhizal truffle‐like fungus *Austrogautieria clelandii*, a known symbiotic partner of *Eucalyptus* spp. (Tedersoo et al., 2010) Scats were collected for four and a half days following dosing (4 hourly for 48 hr, then 6 hourly for the next 36 hr, and then 12 hourly for a further 24 hr) and microscopically examined for the presence of *A. clelandii* spores. Amounts of marker excreted (expressed as proportions of the total number of marker spores seen in fecal samples) and excretion times (estimated as the mid‐point of each collection period) were used to plot the cumulative proportion of spores defecated (a spore gut‐passage curve). Further details of these methods are found in Danks (2012). The first marker spores were observed in scats at 6 and 22 hr after ingestion. No more spores were seen to be defecated after 63 and 69 hr. We use this spore gut‐passage data from Danks (2012) to inform a model of spore dispersal distance.

### 2.3 Modeling spore dispersal distance kernel

We estimate the “dispersal distance kernel” (which describes the probability of spore dispersal to different distances) based on the modeled cumulative probability of spore defecation and empirical observations of swamp wallaby movements (Nathan et al., 2012). We fit separate logistic models to the cumulative proportion of spores defecated by the two wallabies, as estimated by Danks (2012), using the nls function in R (R Core Team, 2019). We then calculated the displacement of tracked wallabies over 69 hr (the maximum time between ingestion and defecation observed by Danks (2012)). From each tracking period, the first 60 min of location data were discarded to allow a return to normal movement patterns after handling. We then extracted all possible 69‐hr long periods. Tracking periods varied in length and sampling intensity; thus, we have more confident predictions of displacement for animals for which we had more tracking data than animals for which we had less tracking data. In each, we multiplied the displacement at each time of recorded location by the probability of spore defecation at that time. We did this separately for the two predicted probabilities of defecation, then took their mean. Finally, we took the mean of the displacements for each of the six tracked wallabies at each time.

This model presents a simplified view of spore dispersal by swamp wallabies; assumptions of the model include (a) truffle‐like fungi sporocarps occur and are ingested evenly across the home range, (b) swamp wallabies use all parts of the landscape surrounding the theoretical point of ingestion, and (c) only time since ingestion determines the predicted distance to which a spore is transported before excretion.

## 3 RESULTS

The animal movement data set contained 4,613 positions, ranging from 126 to 939 positions among tracking periods. Tracking periods ranged from 85 to 544 hr in length (mean ± *SD* = 200.9 ± 144.6 hr), with a combined total of 1,807.9 tracking hours. Location fix interval within tracking periods ranged from 3 min to 26.7 hr, with a mean interval of 23.56 min.

**Figure 1:**
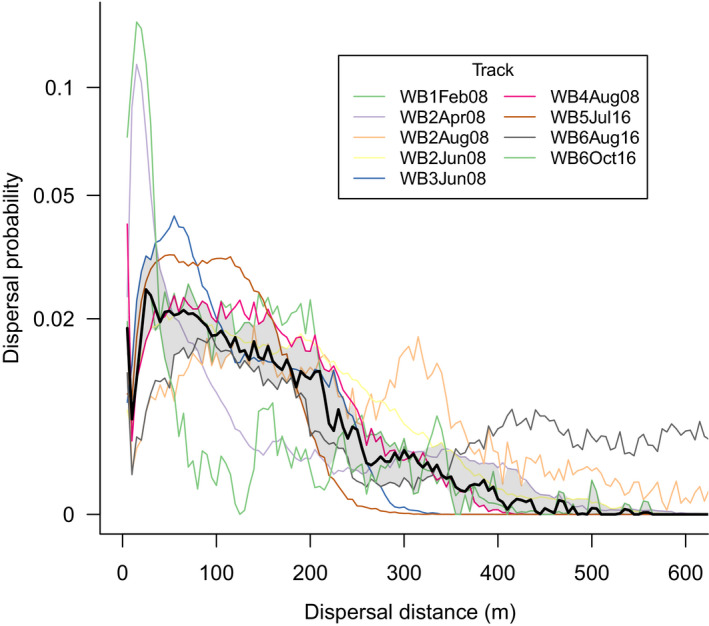
The probability of spore dispersal by each of the tracked wallabies. Thin colored lines indicate the predicted dispersal distances for each of the eight tracked wallabies. Thick black line shows the mean prediction, together with a 50% confidence interval shown as a gray band. Note that the *Y*‐axis is log‐transformed for clarity

**Figure 2:**
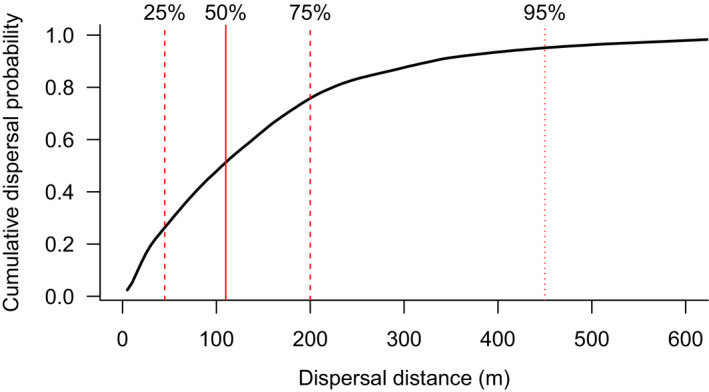
The cumulative probability of dispersal. The overall predicted cumulative dispersal distance for fungal spores ingested by swamp wallabies. The solid vertical line indicates the median dispersal distance, the dashed lines give the 25% and 75% confidence intervals, and the dotted line gives the 95% confidence interval. Note that the *X*‐axis is truncated for clarity

Within the 69‐hr ingestion‐defecation window, the probability of dispersal tended to decrease with increasing distance from the point of ingestion; most spores are predicted to be defecated relatively close to the point of ingestion (Figure 1). Overall, the median predicted distance of spore dispersal by swamp wallabies is 110 m, while most spores (95%) are predicted to be dispersed <450 m from the point of ingestion (Figure 2). Twenty‐five per cent of spores are predicted to be dispersed <45 m and 75% of spores are predicted to be dispersed <200 m (Figure 2). An overall maximum dispersal distance of 1,265 m is predicted.

## 4 DISCUSSION

This study is the first to model the distance to which spores are dispersed by a mycophagous mammal and provides data on ecosystem functions that are poorly known. We hope this model will be a first step toward additional methodological developments similar to those used to examine animal‐mediated seed dispersal. These methods could be routinely employed for the investigation of fungal spore dispersal. Bruns (2019) points out that although the science of fungal ecology is currently where plant ecology was 100 years ago, there are well‐developed theoretical frameworks that could be applied to advance fungal ecology. Practical methods such as the model in this study are vital to more fully understanding the role fungi play in ecosystem functions around the world.

Our model shows that swamp wallabies are highly likely to disperse truffle‐like fungi spores hundreds of meters (and on rare occasions over 1,200 m) from points of ingestion or locations of “parent” sporocarps; these are greater distances than truffle‐like fungi are expected to spread vegetatively through the soil matrix. We estimate that swamp wallabies disseminate spores across distances that would represent LDD for a fungal individual or genet; EM fungal genets typically span <1 to >40 m (Beiler et al., 2010; Bergemann & Miller, 2002; Bonello et al., 1998; Burchhardt et al., 2011; Dahlberg & Stenlid, 1994; Gherbi et al., 1999; Kretzer et al., 2004, 2005). As users of a range of forested habitats, including regenerating forests (Di Stefano et al., 2009), and as one of a small number of vertebrate mycophagists in the study area that consume a high diversity of EM fungi (Nuske et al., 2017), swamp wallabies provide a key ecosystem function by spreading spores between fungal genets.

Our model estimates primary spore dispersal from a single point of origin. Total dispersal kernels (TDKs; Muller‐Landau et al., 2003; Nathan, 2007) more comprehensively describe dispersal by accounting for movement of all dispersal vectors and combining individual dispersal kernels. TDKs can be described at the level of individuals, populations, species, or communities (Rogers et al., 2019). Stephens and Rowe (2020) used a quantitative dispersal network approach, incorporating animal abundance and dispersal effectiveness, to investigate the relative importance of co‐occurring rodents as spore dispersers. Since multiple vectors operate at multiple scales and with varying effectiveness, the complexity of the dispersal of fungi in our study system could be better captured if the contributions and interactions of all biotic and abiotic vectors were included. Unfortunately, knowledge of fungal spore dispersal in the study system is incomplete so we can only hypothesize that numerous other organisms (e.g., birds, reptiles, insects, gastropods, and other mammal species) are contributing to the movements of spores. The movement of spores through food webs (i.e., secondary and further dispersal) is an important knowledge gap in animal‐mediated spore dispersal (Vašutová et al., 2019). Inclusion of more ecological information and data on the effectiveness of each dispersal vector would further refine the accuracy of this model.

Characteristics of the fungal spore could be expected to influence dispersal; however, little is known about relationships between spore morphology and animal‐mediated dispersal. Some authors have speculated that particular spore morphology traits (such as ornamentation, large size, and thick walls) facilitate dispersal to deeper soil layers by invertebrate vectors (Calhim et al., 2018; Halbwachs et al., 2015). Fungal spore morphology varies by taxa (Largent et al., 1980), and it is not known how—if at all—morphology influences gut‐retention time or spore viability; additional experimental data on the passage of a range of commonly consumed taxa through the gut would make the model more comprehensive. Pringle et al. (2015) highlight that adaptive significance and relationship to ecological niche is unknown for most spore and sporocarp morphologies. More research is required to document truffle‐like fungi spore traits and dispersal mechanisms and elucidate their relationships.

Spatial heterogeneity affects animal‐mediated spore dispersal, as animals do not move evenly through the landscape (Johnson et al., 1992). Movement patterns inevitably vary depending on the habitat requirements of different species as well as the health, age, and sex of an individual. Other factors such as availability of water sources, roads, predator presence, and intraspecific competition for resources and mates may all impact the movement patterns of a species or individual (May & Norton, 1996). Some individuals in resource‐rich areas or during good seasons may have limited movements, but in poor seasons the same individual may move much more widely. Swamp wallabies feed throughout the diel cycle and throughout their home ranges. At a fine scale, habitat selection differs between diurnal and nocturnal periods, although food resource availability is a constant factor influencing habitat selection (Di Stefano et al., 2009; Swan et al., 2009). A longer‐term data set collected in a larger range of swamp wallaby habitats would further refine the accuracy of our model.

In assessing spore dispersal distance, we have assumed that the probability of defecation is equal across the area traversed by a swamp wallaby; but this is unlikely to be the case. Spatial and temporal patterns of fecal pellet deposition are related to feeding behavior and movement patterns. Swamp wallaby feeding behavior is not random nor evenly distributed across a landscape; Orlando et al. (2020) found that swamp wallaby foraging patterns were shaped by odor cues. Tammar wallabies (*Macropus eugenii*), eastern gray kangaroos (*M. giganteus*), and red‐necked wallabies (*M. rufogriseus*) have all been shown to defecate at a greater rate while feeding than while resting, and rates vary with age, sex, and season (Johnson et al., 1987; Southwell, 1989; Warner, 1981). These findings are likely also true for swamp wallabies, and pellet deposition would vary spatially, temporally, and among individuals; thus, spore dispersal is not even across their home range. By defecating in the areas in which they feed, swamp wallabies are inadvertently inoculating that area with fungi; in turn, these fungi will benefit not only future populations of swamp wallabies but also the associated plant communities inhabiting that same area.

Landscape‐scale studies of spore dispersal and viability are fundamental in linking dispersal to ecological patterns (Peay et al., 2008), and unfortunately, the basic ecology of many aspects of mammal‐fungi‐plant interactions remains poorly understood. Occurrence of sporocarps in the soil and animal diets are yet to be studied in many EM‐dominated landscapes. In contrast, differences in dispersal distance and disperser effectiveness between species have been studied among frugivorous bird and seed‐harvesting ant assemblages. Any further examination of mammal‐mediated fungal spore dispersal should consider the relatively large body of literature produced on seed dispersal (e.g., Cousens, Hill, French, & Bishop, 2010; Jones & Muller‐Landau, 2008; Lehouck et al., 2009; Levin et al., 2003; Levey et al., 2008; Muller‐Landau et al., 2008; Nield et al., 2020; Spiegel & Nathan, 2007; Will & Tackenberg, 2008).

Despite the enhancements that could be made to our model, our study advances our understanding of animal‐mediated fungal spore dispersal. Our model indicates that swamp wallabies transport spores to distances which promote the spread of the EM fungi they consume. As empirical data about swamp wallabies and other spore dispersing animals around the world accumulates, it will become possible to apply and expand this model to make more robust predictions about animal‐mediated spore dispersal and its implications for ecosystems.

## CONFLICT OF INTEREST

The authors declare that there is no conflict of interest.

## AUTHOR CONTRIBUTION

**Melissa Danks:** Conceptualization (lead); Data curation (equal); Formal analysis (equal); Funding acquisition (equal); Investigation (lead); Methodology (equal); Writing‐original draft (lead); Writing‐review & editing (lead). **Natalie Simpson:** Conceptualization (equal); Data curation (equal); Investigation (equal); Writing‐review & editing (equal). **Todd F. Elliott:** Conceptualization (equal); Investigation (equal); Writing‐review & editing (equal). **Timothy Paine:** Data curation (equal); Formal analysis (equal); Methodology (lead); Software (lead); Writing‐review & editing (equal). **Karl Vernes:** Conceptualization (equal); Funding acquisition (lead); Methodology (equal); Supervision (lead); Writing‐review & editing (equal).

## Data Availability

Animal movement tracking data, cumulative spore excretion data (Danks, 2012), and analysis script: Dryad https://doi.org/10.5061/dryad.08kprr50t
